# LPS Induces Preeclampsia-Like Phenotype in Rats and HTR8/SVneo Cells Dysfunction Through TLR4/p38 MAPK Pathway

**DOI:** 10.3389/fphys.2019.01030

**Published:** 2019-08-27

**Authors:** Minghua Fan, Xiaobing Li, Xiaolin Gao, Lihua Dong, Gang Xin, Liqun Chen, Jianqing Qiu, Yongping Xu

**Affiliations:** ^1^ Department of Obstetrics and Gynecology, The Second Hospital of Shandong University, Jinan, China; ^2^ Department of Laboratory Medicine, Institute of Basic Medicine, Shandong First Medical University and Academy of Medical Sciences, Jinan, China; ^3^ Department of Nephrology, First Affiliated Hospital of Chongqing Medical University, Chongqing, China

**Keywords:** preeclampsia, lipopolysaccharide, toll-like receptor 4, p38 MAPK, spiral artery, inflammation

## Abstract

Accumulating evidence has shown that preeclampsia (PE) was associated with an aberrant maternal-fetal inflammatory response. In the present study, we first found that in human PE placentas levels of toll-like receptor 4 (TLR4), phosphorylated p38 mitogen-activated protein kinase (p-p38 MAPK) and inflammatory cytokines IL-6 and MCP-1 were significantly upregulated. Next, we demonstrated a notable increase in systolic blood pressure (SBP) and proteinuria in lipopolysaccharide (LPS)-treated pregnant rats and concomitant high levels of TLR4 and p-p38 in these PE-like rat placentas, which led to aberrant overexpression of both IL-6 and MCP-1, as well as deficient trophoblast invasion and spiral artery (SA) remodeling, and these abnormalities were ameliorated by SB203580, a reported inhibitor of p38. *In vitro* we further confirmed that LPS triggered the activation of TLR4/p38 signaling pathway, which promoted trophoblast apoptosis and damaged trophoblastic invasion *via* downstream effectors IL-6 and MCP-1; these mutations were rectified by silencing this signaling pathway. These findings elaborated potential mechanisms that aberrant TLR4/p38 signaling might contribute to PE and LPS-induced PE-like symptom by damaging trophoblast invasion and SA remodeling *via* activating inflammatory cytokines including IL-6 and MCP-1.

## Introduction

Characterized by hypertension and proteinuria, PE is a complex multi-system disease of unknown origin, with potentially life-threatening consequences for both mother and baby (Pauli and Repke, 2015). Although the etiology of this disorder remains to be elucidated, numerous researches indicate that altered immune system response and excessive inflammation may play a crucial role in the development of PE (Laresgoiti-Servitje, 2013; Harmon et al., 2016; Shaw et al., 2016). Reportedly, inflammatory responses at the utero-placental interface were related to aberrant extravillous trophoblast (EVT) invasion and impedance of SA remodeling at placentation in transgenic preeclamptic rat models and human PE (Geusens et al., 2010; Warning et al., 2011; Cotechini et al., 2014; Saito and Nakashima, 2014). However, knowledge regarding inflammatory responses in human PE is limited, albeit fast extending in recent years. Therefore, exploring the underlying mechanisms of PE development is fascinating and urgent.

Toll-like receptors (TLRs) were originally recognized as a central regulator of embryogenesis in Drosophila, and key components of the innate immune system expressed on various immune cells, such as dendritic cells (DCs) (Beg, 2002; Panda et al., 2012). TLRs interlink the innate immune system and adaptive immune responses *via* B and T cells and are involved in maintenance of immunotolerance to increased fetal DNA during normal pregnancy (Panda et al., 2012). Inappropriate TLRs activation might be related to breakdown of these mechanisms and trigger secondary inflammation, endothelial malfunction, and ultimately PE (Panda et al., 2012). TLR4 was first known to induce expression of genes involved in inflammatory responses (Beg, 2002) and in the first line of defense of the innate inflammatory immune system at the maternal-fetal interface. TLR4 was abundantly expressed in the placentas of the patients who suffer from PE, suggesting a critical role in inflammation-induced abnormal placentation and development (Koga and Mor, 2010; Bernardi et al., 2012; Panda et al., 2012). Basal expression of TLR4 was significantly elevated in DCs isolated from women with PE, along with higher levels of cytokines (Panda et al., 2012). Therefore, characterizing the potential role of TLR4 in PE is of great importance.

p38 MAPK (p38) is a member of the MAPK family and centrally involved in diverse cellular processes, including inflammation, cell differentiation, cell growth, and cell death (Sargent et al., 2003), and responsible for the production of inflammatory cytokines and downstream signaling events related to inflammation (Orsi and Tribe, 2008). A wealth of data has indicated that it is also a key regulator of TLR4-triggered inflammatory signaling (Redman, 1991; Orsi and Tribe, 2008). Blocking p38 strongly inhibits the production of major inflammatory cytokines such as IL-6, IL-8, and tumor necrosis factor-α (Reister et al., 1999; Fest et al., 2007). Xiong et al. found that phosphorylation of p38 and JNK were increased in human placental explants when exposed to various PE-associated stresses, including angiotensin II, hypoxia and inflammatory cytokines, implying a potential involvement of p38 in PE (Noyola-Martínez et al., 2013). However, the potential association between p38 and TLR4 and whether these molecules have any effects on PE in the setting of inflammation and apoptosis require further elucidation.

Inflammatory cytokines produced by placentas and their release into the maternal circulation throughout pregnancy are the highlights of the regulation of fetal and maternal interactions from embryo implantation until birth (Meekins, 1995; Lockwood et al., 2006; Zhou et al., 2017). Several studies have addressed the importance of different cytokine profiles, such as IL-6 and MCP-1, in the activation and recruitment of macrophages at the placental implantation site in early uncomplicated pregnancy and in PE (Johnson and Lapadat, 2002; Kauma et al., 2002). Enhanced synthesis of IL-6 has been reported to affect the interaction between placental trophoblasts and SA endothelial cells during placentation (Thobe et al., 2007; Peroval et al., 2013). MCP-1 has been reported to be raised in PE women and implicated in PE-associated inflammatory responses (Nair et al., 2014). During placentation of PE, increased MCP-1 could locally recruit macrophages from the blood stream across the endothelium (Meng et al., 2013); and reversely, damaged endothelial cells and/or infiltrated macrophages could produce excess MCP-1 during the initiation and development of PE (Xiong et al., 2013; Nair et al., 2014). Hence, it is reasonable to speculate that these cytokines, when produced in excess, might incite placental insufficiency and lead to the onset of PE by interfering trophoblast invasion and SA remodeling, two major steps during placental growth, and to further identify their potential interactions with TLR4 and p38 signaling.

In the present study, we first showed increased levels of TLR4, p-p38, and inflammatory cytokines IL-6 and MCP-1 in human PE placentas. Next, we demonstrated that LPS induced excessive inflammation, restrained SA remodeling, and resulted in PE-like phenotype in rats, during which TLR4/p38 signaling pathway was hyperactivated, along with increased IL-6 and MCP-1 expression. *In vitro* studies further revealed that trophoblast apoptosis and inadequate trophoblast invasion were driven by LPS stimulation in a TLR4/p38 signaling-dependent manner *via* downstream effectors IL-6 and MCP-1. These findings provided evidence for an important role of TLR4/p38 signaling in the pathogenesis of PE.

## Materials and Methods

### Reagents

Anti-TLR4, p38 and p-p38 antibodies were from Abcam (Cambridge, USA). Anti-IL-6 and anti-MCP-1 were purchased from Cell Signaling Technology, Inc. (Danvers, USA). Anti-β-actin antibodies were obtained from Novus Biologicals, Inc. (Littleton, CO, USA). The shRNAs were synthesized by Cyagen Bioscience Inc. (Guangzhou, China). Anti-β-actin antibodies were obtained from Proteintech (Chicago, IL, USA). Secondary antibodies were from Jackson ImmunoResearch Laboratories, Inc. (West Grove, PA, USA), and Dylight 594 conjugated secondary antibodies were from Pierce, Thermo Fisher Scientific Inc. (Rockford, IL, USA). The p38 inhibitor SB203580 was from Selleck (Houston, TX, USA). LPS were purchased from Sigma Chemical Company (St Louis, MO, USA).

### Animal Studies

Forty-five female Sprague-Dawley rats, 10–12 weeks old, weighing 220–250 g, were purchased from the Animal Center affiliated with Shandong University. All animal studies were carried out with the review and approval of the animal care and use committee of Shandong University [ethical approval code KYLL-2017(GJ)A-0046]. Rats were raised in a light- and humidity-controlled room with free access to food and water. Female rats were rendered pregnant by being housed on proestrus with fertile males at a 2:1 ratio overnight. The day on which pregnancy was confirmed by the presence of vaginal spermatozoa was designated as GD 0. Pregnant rats were randomly allocated into three groups: saline-treated pregnant group (*N* = 15), LPS-treated pregnant group (*N* = 15), and LPS-SB203580-treated group (*N* = 15). For saline or LPS injection, 2 ml of either saline or LPS solution (1 μg/kg, santa cruze, Inc.,) was applied through an infusion pump into the tail vein (infusion rate, 2 ml/h) on GD 14. The LPS-SB203580-treated group additionally received intraperitoneal injection with SB203580 (Calbiochem, La Jolla, CA, USA; 15 mg/kg/day in 200 μl DMSO) once a day from GD 14 till rats were sacrificed. SBP was measured throughout pregnancy by tail cuff plethysmography (Apollo 179; IITC, Woodland Hills, Calif., USA). Urine was collected on GD 13 and 19 for the determination of urinary protein after the rats were weighed and placed in metabolic cages for 24 h, with free access to food and water. On GD 20, rats were sacrificed; their placentas and kidneys were harvested for following experiments.

### Placenta Collection

The experiment was approved by the Clinical Research Ethics Committee of The Second Hospital of Shandong University, and informed consents were obtained from all participants according to the Declaration of Helsinki [ethical approval code KYLL-2017(GJ)P-0019]. A total of 110 subjects, who were pregnant, were recruited from outpatient and inpatient services from the Department of Obstetrics and Gynecology of The Second Hospital of Shandong University from June 2013 to December 2016. In this study, there were 62 pregnant women with PE at 37 ± 4 weeks of gestation (average age 29.5 years) and the same period of healthy pregnant women at 38 ± 1 weeks of gestation (average age 28.1 years) (Table 1). All of the placentas were obtained immediately after cesarean deliveries. The parts of freshly obtained placentas were snap-frozen immediately for processing, fixed with 10% formalin overnight, and embedded in paraffin for immunohistochemistry studies. There were no other obstetric complications in the groups of pregnant women. The diagnosis criteria for PE were SBP ≥ 140 mmHg, diastolic blood pressure (DBP) ≥ 90 mmHg, presence of proteinuria in a 24 h urinary sample exceeding 300 mg, a urine protein/creatinine ratio > 0.3, and ≥ 30 mg/dl protein in a random urine sample (1+ reaction on a standard urine dipstick) (ACOG Committee on Practice Bulletins—Obstetrics, 2002). Pregnant patients with renal disease, hypertension before pregnancy, placental abruption or placenta praevia, anemia or other hematological disease, gestational diabetes, or fetal distress syndrome was excluded from our study.

**Table 1:** Clinical characteristics of the study samples.

	Normal (*n* = 48)	PE (*n* = 62)	*p*
Maternal age (year)	28.1 ± 4.3	29.5 ± 3.7	0.0695
Gestation age at delivery (week)	38 ± 1	37 ± 4	0.0939
Systolic blood pressure (mmHg)	113.20 ± 7.35	163.82 ± 27.26	^*^<0.0001
Diastolic blood pressure (mmHg)	79.54 ± 8.75	106.89 ± 13.76	^*^<0.0001
Proteinuria (g/24 h)	–	3.4 ± 2.5	–

The data are shown as the mean ± s.d. PE, preeclampsia. *p < 0.01 vs. normal pregnancy.

### Cell Culture

HTR-8/SVneo cells, an immortalized cell line that is a well-established model of first trimester human trophoblasts, were grown in RPMI-1640 media supplemented with 10% fetal bovine serum (FBS) (HyClone, USA), 100 U/ml penicillin, and 100 ng/ml streptomycin at 37°C in 5% CO_2_ as previously described (Graham et al., 1993) (Gibco BRL/Invitrogen, USA). To determine the effects of LPS on HTR-8/SVneo cells, LPS (200 ng/ml) was added to the culture medium for 48 h.

### RNA Interference

Plasmid vectors that target TLR4 (3′-GCCACCTCTCTACCTTAATAT-5′) (TLR4 shRNA), p38 (3′-CCATGTTCAGTTCCTTATCTA-5′) (p38 shRNA), and a negative control sequence (3′-CCTAAGGTTAAGTCGCCCTCG-5′) (scrambled shRNA) were purchased from Cyagen Biosciences Inc. (Guangzhou, China). Transfection of HTR8/SVneo cells was performed (1 × 106 cells/well in a 6-well plate) with the indicated plasmids using Lipofectamine 2000 reagent (Invitrogen, Life Technologies Corporation, CA, USA). Western blot analysis and real-time reverse transcriptase PCR were used to validate the efficiency of TLR4 and p38 gene knockdown. After a 24-h incubation with TLR4 shRNA, p38 shRNA or scrambled shRNA, HTR8/SVneo cells were then either treated with or without LPS (200 ng/ml) for an additional 48 h.

### RNA Extraction and Quantitative Real-Time PCR

Real-time reverse transcriptase PCR was used to detect the gene expression in HTR-8/SVneo cells and placental tissues. Total RNA was extracted from the HTR-8/SVneo cells or placental tissues using the RNAiso Plus reagent (Takara, Dalian, China) according to the manufacturer’s instructions. RNA samples were qualified by measuring optic absorbance at 260 and 280 nm with a resultant A260/A280 ratio that ranged from 1.8 to 2.0, which indicated a high purity of the extracted RNA. The concentration of total RNA was calculated on the bases of A260. Aliquots of total RNA from each sample were reverse transcribed into cDNA according to the instructions of PrimeScript™ RT reagent Kit with gDNA Eraser (Perfect Real Time), and then cDNA was used as a template for the PCR reactions, using gene-specific primer pairs. Amplification was performed using SYBR Premix Ex Taq Kit in the LightCycler 480 Real-Time PCR system (Roche Applied Science, Penzberg, Germany). Then Ct values were used to calculate the relative expression level of target mRNAs that were normalized to β-actin. All qRT-PCR experiments were performed in triplicate, including no-template controls. The primers were purchased from Sangon Biotech Co., Ltd. (Shanghai, China). The sequences were designed as follows (Table 2).

**Table 2:** Primers for real time PCR.

Genes	Primers	Sequences
TLR4	Sense	5′-GTTTGAGCCGCAGAAGTATGA-3′
(Human)	Antisense	5′-TCTAAACCAGCCAGACCTTGA-3′
IL-6	Sense	5′-TTCGGTCCAGTTGCCTTCT-3′
(Human)	Antisense	5′-GGTGAGTGGCTGTCTGTGTG-3′
MCP-1	Sense	5′-ATCAATGCCCCAGTCACCT-3′
(Human)	Antisense	5′-TCCTGAACCCACTTCTGCTT-3′
β-actin	Sense	5′-TGACGTGGACATCCGCAAAG-3′
	Antisense	5′-CTGGAAGGTGGACAGCGAGG-3′
IL-6	Sense	5′-CGGAGAGGAGACTTCACAGAG-3′
(Rat)	Antisense	5′-ATTTCCACGATTTCCCAGAG-3′
MCP-1	Sense	5′-TCTGTGCTGACCCCAAGAA-3′
(Rat)	Antisense	5′-TGTGGAAAAGGTAGTGGATGC-3′

### Western Blot Analysis

The total and nuclear protein was extracted from the placental tissues and HTR-8/SVneo cellsusing CelLytic MT Cell Lysis Reagent and NuCLEAR Extraction Kit (Sigma, USA). The protein concentrations were determined using the Bradford assay (Bio-Rad Laboratories, USA). The absorbance was measured at 595 nm using a Beckman DU-640 spectrophotometer (Beckman, Fullerton, CA). Equal amounts of lysate protein (30 μg) were separated on a 10% SDS-PAGE and were wet-transferred onto a polyvinylidene fluoride (PVDF) membrane (Millipore, USA). The immunoblot was performed using primary antibody against the following target proteins: TLR4 (1 μg/ml), p38 (1 μg/ml), p-p38 (1 μg/ml), IL-6 and MCP-1 (5 μg/ml), and β-actin (1 μg/ml). After three washes with PBS (NaCl 9.0 g/L, KCl 0.224 g/L, Na_2_HPO_4_·12H_2_O 3.58 g/L, and NaH_2_PO_4_·2H_2_O 0.28 g/L), the membranes were incubated with horseradish peroxidase (HRP) conjugated secondary antibody (1:10,000) for 1 h at room temperature. Using the ECL system (Amersham Biosciences, USA) and the Bio-Rad electrophoresis image analyzer (Bio-Rad, USA), specific bands were detected. Labworks image analysis software was used to analyze the intensity of band with β-actin as an internal control.

### Co-Immunoprecipitation

Co-IP was performed using Universal Magnetic Co-IP Kit (54002, Active Motif). Briefly, cells were collected using ice-cold PBS/inhibitors buffer. Antibody-cell lysate mixture was prepared by adding 400 μg cell extract and 5 μg TLR4 or p38 antibodies in 500 μl Co-IP/wash buffer in microcentrifuge tubes and was incubated for 4 h at 4°C. Magnetic beads were added to each tube and incubated for 1 h at 4°C with rotating, and washed four times with 500 μl Co-IP/wash buffer. Beads were then resuspended in 2 × reducing loading buffer, heated for 5 min at 95°C, run in a 10% SDS-PAGE gel and subjected to western blot.

### Histology and Immunostaining

Placenta and kidney tissue samples were fixed in a series of 10% formaldehyde, dehydrated graded alcohols, and embedded in paraffin. Placenta and kidney tissue sections (6 mm) were de-waxed, hydrated, and quenched with 0.3% H_2_O_2_ for 30 min. Kidney slices were stained with hematoxylin-eosin (HE) and periodic acid-Schiff (PAS). Then heat-induced antigen retrieval was accomplished, and the slides were incubated with PAS (a fibrinoid tissue marker), the anti-α-SMA antibody (1 μg/ml), and anti-cytokeratin antibody overnight at 4°C. After washing, the sections were incubated with HRP-labeled goat anti-rabbit immunoglobulin for 1 h at room temperature. Staining was completed by incubation with diaminobenzidine chromogen solution (DAB) (Santa Cruz; sc-2017). The sections were counterstained with Harris’s hematoxylin (Sigma-Aldrich, USA), dehydrated, and mounted. Corresponding concentrations of anti-IgG served as non-specific controls. Photographs were taken with a microscope (Leica Microsystems, Germany).

### Immunofluorescence

Cells under different conditions were plated into different 6-well plates and fixed in 4% paraformaldehyde for 20 min, followed by 0.3% Triton X-100 for 10 min. After pre-incubation with 10% fetal calf serum to block nonspecific binding, cells were individually incubated with primary antibodies against TLR4 (1 μg/ml) at 4°C overnight. To visualize the primary antibodies, cells were stained with DyLight 549-conjugated secondary antibodies (1:200) and 4, 6-diamidino-2-phenylindole (DAPI) to visualize the nuclei. Images were observed and captured using an inverted phase/fluorescence microscope (Leica Microsystems GmbH, Germany).

### Transwell Invasion Assay

The transwell Matrigel invasion assay was performed as previously described (Fan et al., 2016). Briefly, 50 μl of diluted 1:4 Matrigel (1:4 dilution; BD Biosciences, USA) in serum-free RPMI 1640 medium was added to the upper chambers of 24-well transwell inserts (BD Biosciences, USA) and incubated at 37°C overnight to enable solidification. HTR-8/SVneo cells were harvested from culture plates at a density of 5 × 10^4^ cells/ml and then plated into the upper chambers of transwell inserts coated with or without Matrigel. The lower chambers were filled with 600 μl of RPMI 1640 medium containing 10% FBS. After incubation at 37°C for 24 h, the upper side of the membranes was scrubbed with cotton swabs to remove the non-invading cells, and the membranes were subsequently fixed in 4% paraformaldehyde (Sigma-Aldrich, USA) and stained with 0.2% crystal violet (Sigma-Aldrich, USA). The number of invading cells in 10 random fields on the underside of the membrane was counted by using an inverted microscope at a magnification of 200× (Olympus, USA).

### FACS Analysis of Apoptosis

Annexin V-FITC and PI apoptosis detection kits were used according to manufacturer’s instructions to measure cell apoptosis. Cellular apoptotic rate was analyzed with a FACScan flow cytometer (BD, Biosciences).

### Transmission Electron Microscopy

For TEM analysis, small pieces of rat renal cortex were fixed in 2% glutaraldehyde and 1% osmic acid for 2 h at 4°C. After two washes in PBS, the samples were dehydrated with an ethanol gradient, washed twice with propylene oxide, soaked in ethoxyline resin overnight, and mounted at 60°C for 48 h. Ultrathin sections (70 nm+) were cut with an ultramicrotome and then viewed under H-7500 transmission electron microscope (Hitachi, Tokyo, Japan). Images with a final magnification of approximately 10,000× were obtained.

### Statistical Analysis

Experiments were performed at least three times. Values were reported as mean ± s.d. Data were analyzed using SPSS 13.0 software. Statistical significance was assessed using ANOVA and LSD-*t*-test. SBP and urinary protein during pregnancy was analyzed by repeated-measures ANOVA. For all statistical tests, *p* < 0.05 or *p* < 0.01 was considered to be statistically significant.

## Results

### Inflammatory Cytokines Were Increased in PE-Bearing Women With High Placental TLR4/p38 Levels

**Figure 1:**
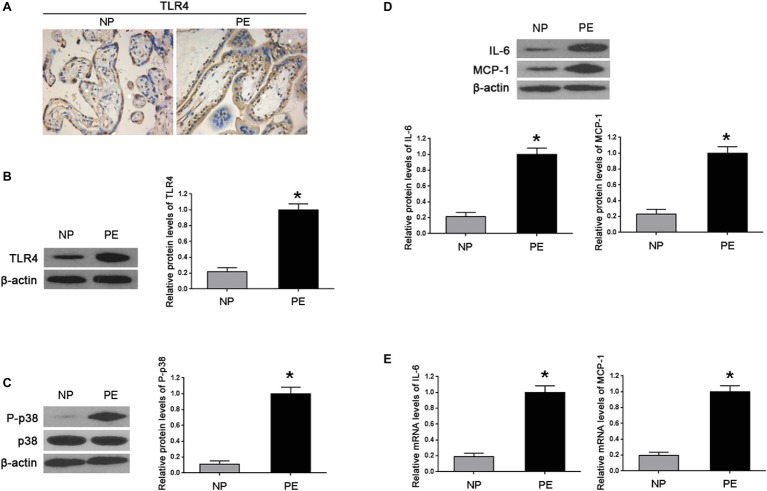
Inflammatory cytokines increased in the placenta of PE-bearing women with high TLR4/p38 levels. **(A)** Immunohistochemistry showed that TLR4 protein staining substantially enhanced in the cytoplasm of PE placenta trophoblasts in comparison with that of normal pregnancy (NP). **(B,C)** Western blot illustrated that the expression levels of TLR4 and p-p38 were markedly increased in the placentas of PE compared with that found during normal pregnancy. **(D,E)** Western blot and real-time PCR showed that both IL-6 and MCP-1 in PE placentas were significantly higher than those of normal pregnancy on protein and mRNA levels. Values denote the mean ± s.d.; ^*^p < 0.01 vs. normal pregnancy. NP, normal pregnancy; PE, preeclampsia.

First, we determined the levels of TLR4 and p-p38 in normal gestational and preeclamptic placental tissues. Immunohistochemistry showed that TLR4 was mainly detected in the cytoplasm of trophoblasts in both normal and preeclamptic placentas; and increased staining of TLR4 was shown in PE (Figure 1A). By western blot, we showed TLR4 and p-p38 protein levels were markedly increased in PE placentas, compared with normal gestational placentas (^*^p < 0.01 vs. NP) (Figures 1B,C). Concomitantly, enhanced protein and mRNA expressions of IL-6 and MCP-1 were revealed in PE, in comparison with normal pregnancy (^*^p < 0.01 vs. NP) (Figures 1D,E).

### LPS Altered the Expression of TLR4 and p-p38 in the Placenta of Rats

**Figure 2:**
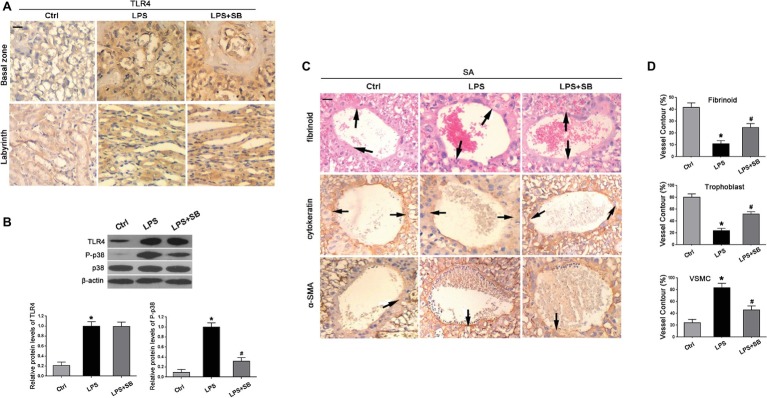
Expressions of TLR4 and p-p38 and their effects on trophoblast invasion and SA remodeling in LPS-treated rats. **(A)** Immunohistochemistry suggested that TLR4 staining was enhanced in the cytoplasm in LPS-treated and LPS plus p38 inhibitor SB203580-treated rats, which was predominantly expressed by spongiotrophoblast cell, trophoblastic giant cell, and glycogen cell in the basal zone as well as trophoblastic epithelium in the labyrinth of rat placenta. Scale bar = 100 μm. **(B)** Western blot revealed that LPS treatment gave rise to TLR4 expression and p38 phosphorylation in the placenta of pregnant rats. The addition of SB203580 dramatically abrogated p38 activation, whereas showed little alterations on TLR4 expression. Values denote the mean ± s.d.; ^*^p < 0.01 vs. control, ^#^*p* < 0.01 vs. LPS-treated group. **(C)** SAs from all groups exhibited evidence of remodeling, including the endovascular trophoblast cells of cytokeratin staining (arrows) resting on a fibrinoid layer of PAS staining (arrows) and the smooth muscle cells of α-SMA staining (arrows). Scale bar = 100 μm. **(D)** The percentages of trophoblast, fibrinoid, and VSMC of total SA contour length were determined. Values denote the mean ± s.d.; ^*^p < 0.01 vs. control, ^#^*p* < 0.05 vs. LPS-treated group. Ctrl, control group; SB, SB203580.

To further identify the roles of TLR4 and p38 in the pathogenesis of PE, PE-like rat models were established, by randomly allocating pregnant rats into three groups who correspondingly underwent saline, LPS, or LPS + SB203580 injection. The immunohistochemistry results showed that TLR4 staining was significantly increased in the cytoplasm of spongiotrophoblast cell, trophoblastic giant cell, and glycogen cell in the basal zone, as well as trophoblastic epithelium in the labyrinth in LPS-treated rat placenta, whereas its staining was not affected by SB203580 treatment (Figure 2A). Through western blot, we found that LPS exposure gave rise to the expression of TLR4 and p-p38 compared with the saline injection controls (^*^p < 0.01 vs. Ctrl, ^#^*p* < 0.01 vs. LPS) (Figure 2B), in consistent with findings that TLR4 and p-p38 protein levels were elevated in human PE samples.

### Inhibition of p38 Ameliorated Hypertension and Proteinuria in LPS-Induced PE-Like Rats

**Figure 3:**
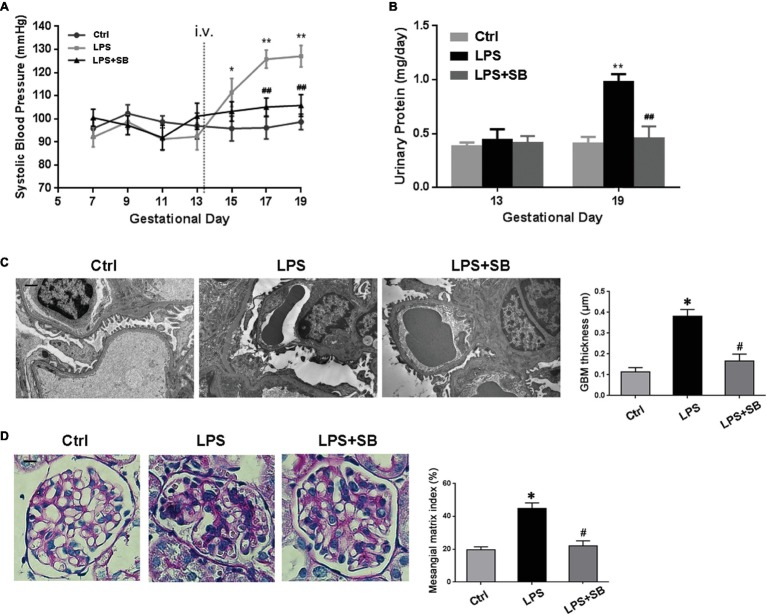
Effects of p38 inhibition on the hypertension and proteinuria of LPS induced PE-like rats and the morphological features of their kidneys. **(A,B)** The SBP and urinary protein were presented, respectively. No remarkable alterations in SBP and 24 h urinary protein levels were observed before LPS infusion among different groups. After injection, SBP levels of LPS-treated rats were much higher and were prohibited and restored by SB203580 from GD 17. The 24 h urinary albumin excretion was significantly higher in LPS-treated group after LPS infusion, and these abnormalities were essentially prevented by treatment of SB203580. Values denote the mean ± s.d.; *p* > 0.05 vs. control before LPS infusion, ^*^p < 0.05, ^**^p < 0.01 vs. control on the corresponding GD after LPS infusion. ^##^*p* < 0.01 vs. LPS-treated group on the corresponding GD after LPS infusion. **(C)** Kidney ultrastructure on TEM in different groups. GBM thickening and foot process effacement were demonstrated in LPS group, which were attenuated in LPS-p38 inhibitor group. Scale bar = 1 μm. **(D)** Mesangial and glomerular area by PAS staining. There occurred moderate mesangial proliferation, GBM thickening, and adhesion of the glomerular tuft to the Bowman’s capsule in LPS-treated group, indicative of advanced renal injury. These mutations were attenuated in LPS-p38 inhibitor group. Values denote the mean ± s.d.; ^*^p < 0.01 vs. control, ^#^*p* < 0.01 vs. LPS-treated group. Scale bar = 100 μm. Ctrl, control group; SB, SB203580.

As PE is characterized with hypertension and proteinuria, SBP and urinary protein were determined. Results showed that there were no significant differences on the SBP values among the three groups before any injection (*p* > 0.05) (Figure 3A). After injection, SBP levels of LPS-treated rats were much higher than controls from GD 15 to the GD they were sacrificed (^*^p < 0.05, ^**^p < 0.01 vs. control on the corresponding GD after LPS infusion. ^##^*p* < 0.01 vs. LPS-treated group on the corresponding GD after LPS infusion) (Figure 3A). High levels of SBP were prohibited and restored by SB203580 from GD 17 (^##^*p* < 0.01 vs. LPS) (Figure 3A). The 24 h urinary albumin excretion of rats showed no difference among different groups prior to LPS infusion (*p* > 0.05) (Figure 3B), but the level was significantly higher in LPS-treated group after LPS infusion (^**^p < 0.01 vs. control on the corresponding GD after LPS infusion) (Figure 3B). These abnormalities were essentially prevented by treatment of SB203580 (^##^*p* < 0.01 vs. LPS-treated group on the corresponding GD after LPS infusion) (Figure 3B). Concomitantly, SB203580 pretreatment did not significantly impact TLR4 expression (*p* > 0.05) (Figure 2B), albeit distinctly abolished p38 activation (^#^*p* < 0.01 vs. LPS) (Figure 2B) as was shown by western blot.

### Inhibition of p38 Prevented the LPS-Induced PE-Like Rats From Renal Injury

Next, we investigated the severity of kidney injury in LPS-induced PE-like rats by transmission electron microscopy (TEM) and PAS staining. On TEM, there were a significant thickening of the glomerular basement membrane (GBM) and a markedly narrowed residual glomerular capillary lumen resulted from swollen endothelial cell in LPS-treated group, which were both ameliorated by treatment with SB203580 (^*^p < 0.01 vs. Ctrl, ^#^*p* < 0.01 vs. LPS) (Figure 3C). PAS staining showed that the glomerular tufts are enlarged, swollen, and solidified, and the PAS-positive matrix was prominent in LPS-treated group (Figure 3D). To determine the alteration of mesangial matrix, we evaluated the mesangial/glomerular area and found it significantly increased in LPS-treated group, which markedly reduced by SB203580 (^*^p < 0.01 vs. Ctrl, ^#^*p* < 0.01 vs. LPS) (Figure 3D).

### Inhibition of p38 Ameliorated Deficient Trophoblast Invasion and Impaired SA Remodeling in LPS-Treated Rats

Immunohistochemistry and PAS revealed the evidence of SA remodeling in control rats, which was characterized by cytokeratin-positive trophoblast cells resting on a fibrinoid layer and the absence of vascular smooth muscle cell (VSMC) marker α-smooth muscle actin (α-SMA) (Figure 2C). Compared with the control, LPS treatment resulted in faint staining of cytokeratin, decreased deposition of fibrinoid, and enhanced staining of α-SMA, illustrating that trophoblast invasion of SA was impeded in LPS-treated pregnant rats (Figure 2C). These abnormalities were partially reversed by pretreatment of SB203580, indicative of enhanced staining of cytokeratin and fibrinoid wall, but weakened staining of α-SMA (Figure 2C). In addition, the percentages of fibrinoid and trophoblast of total SA contour length were notably declined (^*^p < 0.01 vs. Ctrl, ^#^*p* < 0.05 vs. LPS) (Figure 2D); nevertheless, an increased percentage of VSMC contour length was observed in LPS rats in comparison with control group (^*^p < 0.01 vs. Ctrl) (Figure 2D), which were both prevented by pretreatment of SB203580 (^#^*p* < 0.05 vs. LPS) (Figure 2D).

### Inhibition of p38 Attenuated Over-Expression of Inflammatory Cytokines in the Placenta and Kidney of LPS-Treated Rats

**Figure 4:**
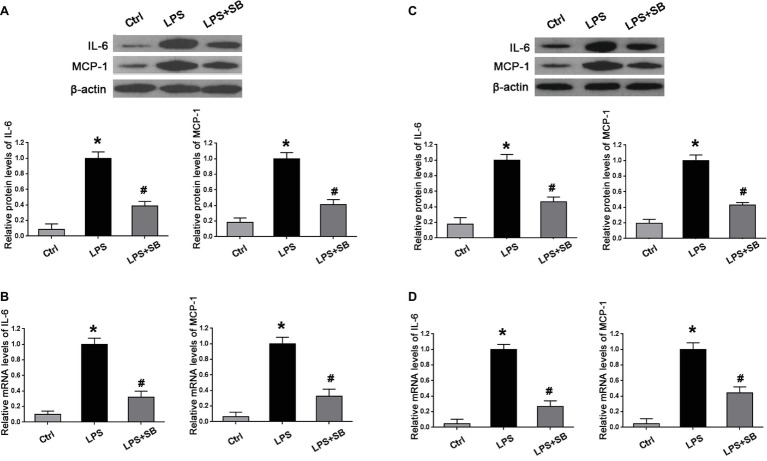
Alterations of inflammatory cytokines expression in the placenta and kidney of different pregnant groups in rats. Western blot and real-time PCR presented that LPS treatment gave rise to the expressions of inflammatory markers IL-6 and MCP-1 on both protein and mRNA levels in **(A,B)** placentas and **(C,D)** kidney cortex; aberrant expressions of these two proteins and mRNA were abolished by pretreatment of SB203580. Values denote the mean ± s.d.; ^*^p < 0.01 vs. control, ^#^*p* < 0.05 vs. LPS-treated group. Ctrl, control group; SB, SB203580.

We further determined the expression of inflammatory markers, including IL-6 and MCP-1 in the placentas and kidneys by western blot and real time PCR. As expected, LPS treatment significantly increased the production of IL-6 and MCP-1 in the placenta (^*^p < 0.01 vs. Ctrl) (Figures 4A,B) and kidney (^*^p < 0.01 vs. Ctrl) (Figures 4C,D) in comparison with control group; SB203580 pretreatment could partially reduce the expressions of their proteins and mRNA in the placenta (^#^*p* < 0.05 vs. LPS) (Figures 4A,B) and kidney (^#^*p* < 0.05 vs. LPS) (Figures 4C,D) compared with LPS group. These results indicated that p38 might play a crucial role in inflammatory response in LPS-treated pregnant rats.

### TLR4 shRNA Suppressed Over-Activation of p38 and Decreased Excessive Expression of Inflammatory Cytokines in LPS-Treated HTR-8/SVneo Cells

**Figure 5:**
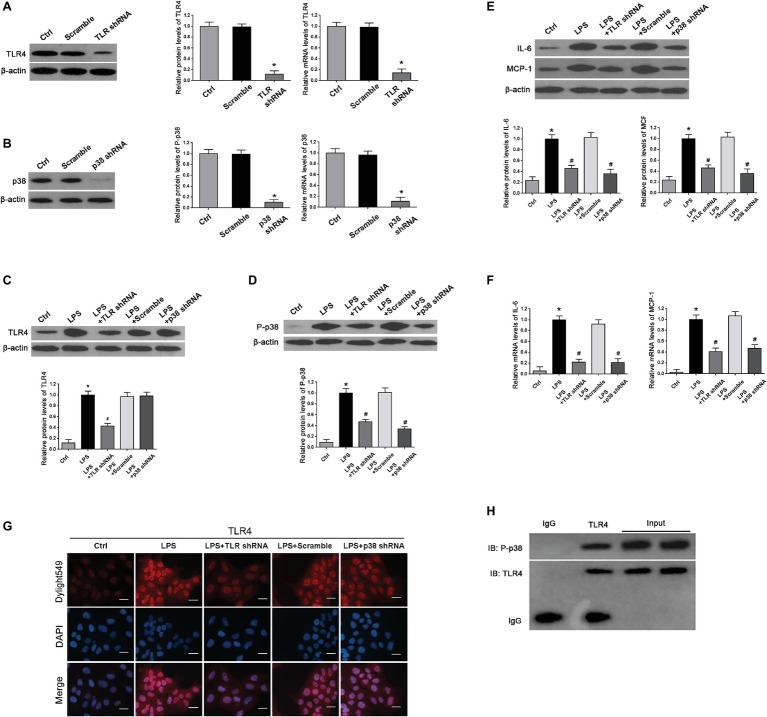
Determination of expression of TLR4, p-p38, and inflammation cytokines and their interplays in cultured HTR-8/SVneo cell. **(A,B)** Both western blot and real-time PCR demonstrated a relatively high knockdown efficiency of TLR4 or p38 gene after shRNA transfection for 24 h. Values denote the mean ± s.d.; ^*^p < 0.01 vs. control. **(C,D)** Western blot demonstrated that LPS treatment promoted TLR4 expression and p38 phosphorylation compared with controls. However, in comparison with LPS group, expressions of TLR4 and p-p38 in LPS-treated cells were significantly decreased by transfection with TLR4 shRNA; p38 shRNA transfection in LPS-treated cells dramatically deleted p38 activation without affecting TLR4 expression. Values denote the mean ± s.d.; ^*^p < 0.01 vs. control, ^#^*p* < 0.01 vs. LPS-treated group. **(E,F)** Western blot and real-time PCR showed that excessive expressions of IL-6 and MCP-1 were induced on both protein and mRNA levels in LPS-treated cells compared with control; these aberrant expressions were abolished by TLR4 or p38 gene knockdown. Values denote the mean ± s.d.; ^*^p < 0.01 vs. control and ^#^*p* < 0.01 vs. LPS-treated group. **(G)** Immunofluorescence staining revealed TLR4 expression in the cytoplasm of the HTR-8/SVneo cells. Compared with control, LPS treatment markedly enhanced TLR4 staining, which was decreased by transfection with TLR4 shRNA. Nevertheless, there were no distinguishable differences on TLR4 staining between p38 shRNA transfection and LPS treatment. Scale bar = 20 μm. **(H)** CO-IP demonstrated the interactions between TLR4 and p38. Cell lysates were immunoprecipitated with anti-TLR4 antibodies and were subjected to immunoblot with the indicated antibodies. Ctrl, control group; Scramble, scrambled shRNA.

To confirm the potential interactions between P38 and TLR4 in LPS-treated HTR-8/SVneo cells, we first validated TLR4 and p38 knockdown efficiency (^*^p < 0.01 vs. Ctrl) (Figures 5A,B). Next, we determined whether activation of TLR4 induced phosphorylation of p38 and thereby led to expression of inflammatory cytokines. We found that the over-expression of TLR4 and p-p38 induced by LPS treatment (^*^p < 0.01 vs. Ctrl) were both reduced by TLR4 shRNA transfection in LPS-treated cells (^#^*p* < 0.01 vs. LPS) (Figures 5C,D); meanwhile, p38 shRNA transfection dramatically abrogated p38 activation (^#^*p* < 0.01 vs. LPS) whereas had little impacts on TLR4 expression in LPS-treated cells (*p* > 0.05) (Figures 5C,D). Moreover, we determined the effects of TLR4 or p38 shRNA on inflammatory markers MCP-1 and IL-6. We found that both MCP-1 and IL-6 were increased in LPS-treated HTR-8/SVneo cells (^*^p < 0.01 vs. Ctrl), which were substantially diminished by TLR4 or p38 gene knockdown on protein and mRNA levels (^#^*p* < 0.01 vs. LPS) (Figures 5E,F). Through use of immunofluorescence, we found that compared with the control, TLR4 was strongly stained in LPS-treated cells, which was weakened after TLR4 gene silencing, and p38 shRNA transfection showed little effects on TLR4 staining (Figure 5G). Furthermore, we demonstrated potential interactions between TLR4 and p38 by Co-IP, and found that reproducibly detectable levels of p38 were present in the anti-TLR4 immunocomplexes, demonstrating an endogenous interaction between these two proteins in HTR-8/SVneo cells (Figure 5H). These results indicated that p38 might play as a downstream effecter of TLR4 after LPS stimulation by promoting expression of IL-6 and MCP-1.

### Inhibition of TLR4/p38 Signaling Pathway Reinstated Invasive Capacity and Restrained Apoptosis in LPS-Treated HTR-8/SVneo Cells

**Figure 6:**
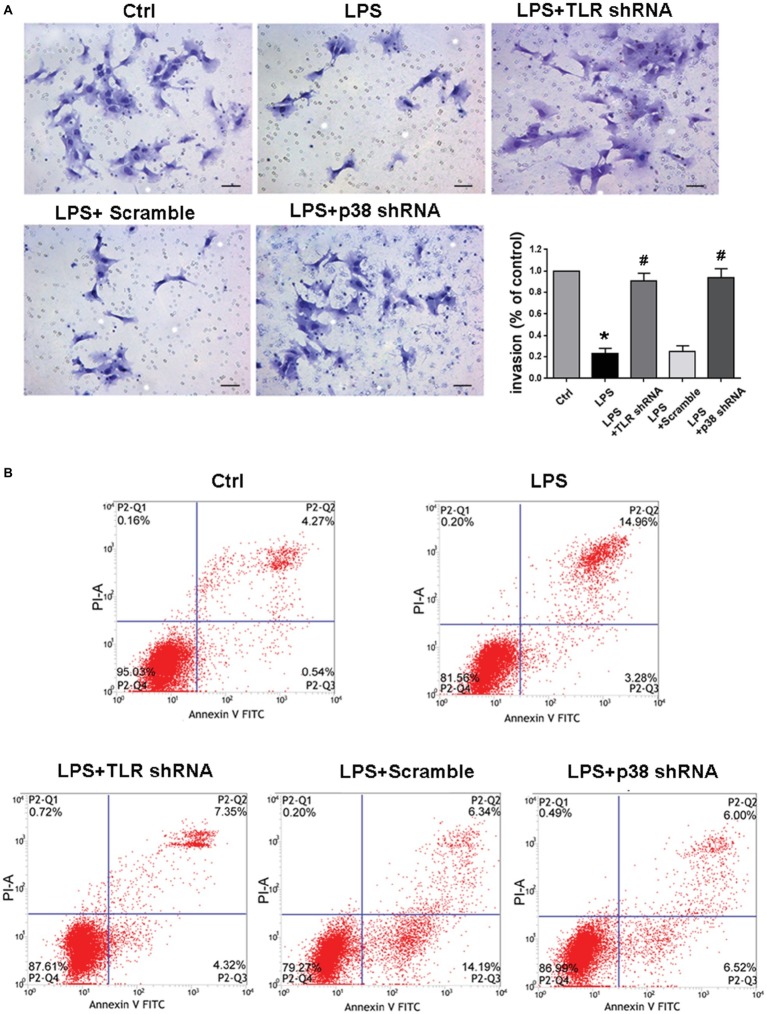
Determining invasion and apoptosis of LPS-treated HTR-8/SVneo cells in different groups. **(A)** Transwell Matrigel invasion assay analyzed HTR-8/SVneo cells invasion, and the results were represented as invasion percentage. In comparison with control, the invasive ability of LPS-treated cells decreased, which was ameliorated by transfection with TLR4 shRNA or p38 shRNA. Values denote the mean ± s.d.; ^*^p < 0.01 vs. control, ^#^*p* < 0.01 vs. LPS-treated group, scale bar = 40 μm. **(B)** The total apoptotic rates in different groups were identified. After LPS treatment for 24 h, the total apoptotic rate was enhanced in HTR-8/SVneo cells, but was reversed by either TLR4 or p38 gene knockdown. Ctrl, control group; Scramble, scrambled shRNA.

To determine the potential impacts of TLR4/p38 signaling on EVT invasion under culture, we performed transwell invasion assays. The EVT invasion was significantly decreased after LPS treatment (^*^p < 0.01 vs. Ctrl), whereas TLR4 or p38 shRNA transfection significantly abolished such diminished EVT invasion (^#^*p* < 0.01 vs. LPS) (Figure 6A). After HTR-8/SVneo cells were challenged with LPS for 24 h, the total apoptotic rate was enhanced, but it was reversed by either TLR4 or p38 gene knockdown (Figure 6B), indicating that LPS-induced PE-like phenotype and inflammatory response were associated with apoptosis of invasive EVTs.

## Discussion

Excessive systemic inflammation at the maternal-fetal interface has been described as an enormously important characteristic of PE. LPS, an endotoxin of gram-negative bacterial cell walls, has been demonstrated to mediate inflammatory processes in association with female reproduction (Dowling et al., 2012). In 1994, Faas et al. (1994) developed a classic rat model for PE by injecting LPS into pregnant rat on gestational day (GD) 14. The rat model exhibited all the symptoms and the placental pathological changes that happened in human PE. In the present study, we generated a similar PE rat model by intravenous injection of LPS and were able to discuss the underlying mechanism to explore the development of PE.

Previous research has demonstrated that LPS, by binding to pattern recognition receptors such as TLR4, activated the maternal innate immune system and increased the production of a range of inflammatory cytokines and chemokines (Koga and Mor, 2010). And there are a number of *in vivo* animal studies that demonstrated bacterial infection related to preterm delivery through TLR4 signaling (Elovitz et al., 2003). However, the impacts of TLR4 signal transduction pathways on PE in the setting of inflammation remain unclear. In the present study, we showed that LPS induced over-expression of TLR4, together with up-regulation of IL-6 and MCP-1 in rat placentas, suggesting that the placenta may recognize pathogens through this receptor, induce downstream inflammatory responses, consistent with our findings in human PE placentas that TLR4 and cytokine profiles were both upregulated and result in ischemic preeclamptic placenta. The ischemic preeclamptic placenta in response synthesized multiple cytokines including TNF-α and IL-6, further complicating the pregnancy (Harmon et al., 2016). Therefore, the observed up-regulation of TLR4 in response to LPS exposure might be a mechanism to augment a cascade of inflammatory response and initiate the progression of PE.

MAPKs have been illustrated to be important mediators of TLR signaling pathways (Thobe et al., 2007; Peroval et al., 2013) and TLR4 contributed to p38 and NF-κB activation in LPS-mediated inflammatory processes in macrophages (Xu et al., 2014). Here in both human and rat PE placentas, significant activation of p38 was observed, which concomitantly occurred with aberrant expression of TLR4 and cytokines IL-6 and MCP-1. Multiple studies have demonstrated a key role of p38 in regulating expression of many inflammation-related genes (Liu et al., 2008). Our study further confirmed a critical role of p38 in inflammatory reactions of PE. p-p38 expression was associated with LPS-induced hypertension and proteinuria excretion, and with increased IL-6 and MCP-1, which were partially reversed by abrogation of p38 activation. In spite of deletion of p38 hyper-activity and attenuation of inflammatory responses, pretreatment of p38 inhibitor did not alter TLR4 expression, suggesting a signaling cascade of TLR4/p38 in response to LPS stimulation. From these, it was plausible to speculate that p38 may act as a downstream activator of TLR4 signaling, and the activation of this signaling may accelerate the development of a preeclamptic state by increasing the levels of inflammatory cytokines. Notably, increasing evidence has shown that proper activation of p38 was also required during normal pregnancy (Mudgett et al., 2000; Daoud et al., 2005), thus delicate regulation, instead of excessive inhibition, of the TLR4/p38 signaling axis should be considered and calls for further investigation.

Studies have shown that inadequate EVT invasion is one of the major characteristics of PE, which could result in subsequent failure of SA remodeling (Straszewski-Chavez et al., 2005; Saito and Nakashima, 2014). EVT cells are a highly migratory cell population that invade the maternal decidua and inner third of the myometrium and remodel the uterine SAs (Brosens et al., 1972; Pijnenborg et al., 2006). EVT has become an important focus of PE research over the years (Brosens et al., 1972). And increasing data have suggested that PE was associated with the sudden onset of widespread apoptosis of invasive EVT in the placental bed, which inhibited EVT invasion into the SA (Straszewski-Chavez et al., 2005). Despite these studies, the molecular mechanisms that regulated EVT invasion and utero-placental SA remodeling remained controversial. In this context, we further explored the potential involvement of TLR4 and p38 in EVT invasion of PE. Our *in vitro* data demonstrated that TLR4 and p-p38 over-activation were stimulated by LPS, with increased inflammatory hallmarks IL-6 and MCP-1 as well as impaired EVT invasion, which could be significantly prohibited by TLR4 gene knockdown. However, the addition of p38 shRNA, although eliminated p38 over-activation, ameliorated the expressions of IL-6 and MCP-1 and preserved EVT invasion, without affecting TLR4 expression. Prior studies illustrated that p38 represented a key biological downstream target of TLR4 in both acute/chronic inflammatory disease and cancers (Li et al., 2014; Meng et al., 2017). Nevertheless, different from those studies, we provided evidence that TLR4/p38 pathway was activated during placentation and contributed to insufficient EVT invasion by triggering the release of downstream inflammatory cytokines in exposure to LPS.

As pointed out above, EVT invasion contributed to the success of maternal SA remodeling. Mounting evidence suggested that aberration in placental vascular modification resulting from shallow EVT invasion was associated with obstetric complications (Kaufmann et al., 2003; van den Brûle et al., 2005; Fan et al., 2016). Our observations in LPS-treated pregnant rats illustrated that the inadequate trophoblast invasion and SA remodeling, as the vital consequence of TLR4/p38 signaling activation, were characterized by reduced cytokeratin-positive trophoblast cells and outer-neighboring fibrinoid layer, and increased α-SMA-positive VSMC; all of these were accompanied by alterations of IL-6 and MCP-1. Also, it has been suggested that inflammatory cytokines produced during PE may contribute to inadequate SA remodeling, deficient placental perfusion, and consequent hypertension (Gadonski et al., 2006). Intriguingly, recent evidence showed that inadequate EVT invasion of SAs initiated ischemia in the placenta, which in turn resulted in an increased release of inflammatory cytokines in the placenta (Gilbert et al., 2008). These observations provided evidence that there might be a unique interaction between inflammatory cytokines and inadequate EVT invasion during PE. Thus, we inferred that inflammatory cytokines were downstream targets of TLR4/p38 signaling pathway, which contributed to the occurrence and development of PE.

Diminished renal plasma flow, decreased GFR, and proteinuria constituted key renal manifestations of PE (Moran et al., 2004). A constellation of findings about glomerular, tubular, and arteriolar changes were noted at the microscopic level in kidneys of women with PE, and the glomerular lesion was generally regarded as a hallmark of PE (Hladunewich et al., 2007). Consistent with prior studies, we found renal abnormalities occurred in LPS-treated pregnant rats. PAS staining displayed expanded mesangial matrix, swollen endothelial cytoplasm, glomerular swelling with occlusion of capillary loops, increases of the glomerular size and glomerular capillary lumen diameters in LPS-treated pregnant rats. TEM further demonstrated the significant thickening of the glomerular basement membrane and the foot process effacement in LPS-treated rats, and these lesions were significantly reinstated by treatment of p38 inhibitor. Notably, although proteinuria is often related to aberrant renal structural changes, small and early renal damage would not necessarily lead to proteinuria. Retraction and fusion of podocyte foot processes were found ahead of the onset of proteinuria in models of kidney diseases (Li et al., 2008; Liu, 2010; May et al., 2014; Lv et al., 2018). In addition, Blair et al. showed some cases classified as early-onset pre-eclampsia (EOPET) did not have documented proteinuria (Blair et al., 2013). Thus, it is interesting to further identify the potential relationship between PE and early kidney injury in future studies.

In conclusion, the present study demonstrated that the activation of TLR4/p38 signaling in response to LPS, which resulted in inadequate trophoblast invasion and SA remodeling, may be a mechanism to augment the inflammatory responses and may contribute to the development of PE; the blockade of this axis may safeguard against PE-like symptoms and prevent the development of PE.

## Data Availability

The raw data supporting the conclusions of this manuscript will be made available by the authors, without undue reservation, to any qualified researcher.

## Ethics Statement

This study was carried out in accordance with the recommendations of “Clinical Research Ethics Committee of The Second Hospital of Shandong University” with written informed consent from all subjects. All subjects gave written informed consent in accordance with the Declaration of Helsinki. The protocol was approved by the “Clinical Research Ethics Committee of The Second Hospital of Shandong University.”

## Author Contributions

MF, JQ, and YX designed experiments, interpreted data, prepared the figures, and wrote the manuscript. XL, XG, and LD performed the experiments and analyzed the data. GX and LC revised the manuscript. All authors approved the final version of the manuscript.

### Conflict of Interest Statement

The authors declare that the research was conducted in the absence of any commercial or financial relationships that could be construed as a potential conflict of interest.
